# Reference Gene Selection for Analyzing the Transcription Patterns of Two Fatty Acyl-CoA Reductase Genes From *Paracoccus marginatus* (Hemiptera: Pseudococcidae)

**DOI:** 10.1093/jisesa/ieab072

**Published:** 2021-10-04

**Authors:** Xiao Liang, Qing Chen, Chunling Wu, Ying Liu, Zhiling Han, Mufeng Wu

**Affiliations:** 1 Environment and Plant Protection Institute, Chinese Academy of Tropical Agricultural Sciences/Key Laboratory of Integrated Pest Management on Tropical Crops, Ministry of Agriculture and Rural Affairs, Haikou, Hainan 571101, China; 2 Sanya Research Academy, Chinese Academy of Tropical Agriculture Science/Hainan Key Laboratory for Biosafety Monitoring and Molecular Breeding in Off-Season Reproduction Regions, Sanya, Hainan 572000, China; 3 College of Plant Protection of Hainan University, Haikou, Hainan 570228, China

**Keywords:** *Paracoccus marginatus*, fatty acyl-CoA reductase gene, reference gene, cassava cultivar, transcription analysis

## Abstract

*Paracoccus marginatus* (Hemiptera: Pseudococcidae), known as the papaya mealybug, could cause considerable yield loss of several plants. To date, there is no molecular-based study of *P. marginatus*. Fatty acyl-CoA reductases (FARs) are key enzymes involved in wax synthesis. In the present study, we cloned and characterized coding sequences (CDS) of two *FAR* genes from *P. marginatus*. The results showed that *PmFAR1* and *PmFAR2* CDS were 1,590 and 1,497 bp in length, respectively, and sequence analysis indicated that these two genes both had the conservative motifs belonging to FAR_C superfamily. Furthermore, seven candidate reference genes were analyzed for their expression stability by using common algorithms including comparative ΔCq method, geNorm, NormFinder, BestKeeper, and RefFinder. Eventually, β*-actin* and *GAPDH* were the best reference genes in evaluating the expression of those two *FAR* genes. We found that *PmFAR1* and *PmFAR2* showed distinct expression patterns in different life stages. Moreover, the transcription of *PmFAR1* and *PmFAR2* in *P. marginatus* fed on resistant cassava cultivars was significantly lower compared with those fed on susceptible ones, indicating the potential function of *FAR* genes in cassava resistance to *P. marginatus.* The present study might help in better understanding the molecular mechanism of cassava resistance to mealybug.

The papaya mealybug, *Paracoccus marginatus* Williams and Granara de Willink (Hemiptera: Pseudococcidae), is a polyphagus insect and a pest of various tropical fruits, vegetables, and ornamental plants (Miller and Miller 2002). *Paracoccus marginatus* is a native species in Mexico and Central America, and now it had spread to 34 countries (Williams and Willink 1992). In China, it had been previously recorded in Taiwan, and firstly found on *Jatropha podagrica* from Xishuangbanna of Yunnan Province (Zhang and Wu 2015). The most common hosts in China include *Carica papaya* L. (papaya), *Manihot esculenta* (cassava), *Citrus* spp. (citrus), *Solanum melongena* L. (eggplant), *Plumeria* spp. (plumeria), and *Acalypha* spp. (acalypha) (Wang et al. 2018). This insect can cause 50–70% of cassava yield loss or even lead to no harvest at all especially in Hainan, Guangxi, and Yunnan Provinces (Wu et al. 2021). 

So far, the use of insecticides is the most common method to control mealybug. However, the body of this mealybug is covered with water-repellent wax which may reduce the control effect of insecticides (Williams and Watson 1988); therefore, some researchers tried to mine and manipulate the potential wax biosynthesis genes. Fatty acyl-CoA reductases (FARs) are key enzymes involved in wax biosynthesis, which play an important role in reducing water evaporation and enhancing defense against microorganisms (Pu et al. 2012, Nguyen et al. 2014). When the cotton mealybug, *Phenacoccus solenopsis*, was treated with the insecticide spirotetramat, the relative expression levels of *PsFAR1* were upregulated and there was no effect on the expression of *PsFAR2*. The authors speculated that *PsFAR1* and *PsFAR2* played different roles in responding to spirotetramat, and *PsFAR1* may contribute to make wax-ester of the insect epicuticle (Li et al. 2016). The males of the beneficial scale insect *Ericerus pela* (Hemiptera: Coccoidea) can secret white wax, and its *EpFAR* gene is mainly expressed in cuticle. Besides, the EpFAR protein was only localized in the wax glands and testis (Hu et al. 2017). However, as far as we know, there is no molecular-based study of *P. marginatus*, and the *FAR* genes and their expression patterns have not been identified yet.

Quantitative reverse transcription–polymerase chain reaction (RT-qPCR) is the most sensitive and accurate method for measuring and validating gene expression (Bustin et al. 2005, VanGuilder et al. 2008, Gao et al. 2017). Internal reference genes are usually applied to normalize quantitative fluorescence data on the target gene which can ensure an accurate detection of gene expression levels. However, increasing evidence has suggested that there are no universal reference genes that are suitable for all experimental conditions in all species (Morales et al. 2016). With the development of large-scale high-throughput sequence technologies to identify genes that are differentially expressed between conditions, finding a reference gene that has stable expression under specific experimental conditions become much more convenient (Arya et al. 2017, Zheng et al. 2019).

To date, studies about reference gene selection in mealybug species were all concentrated in *Ph. solenopsis* (Arya et al. 2017, Singh et al. 2019, Zheng et al. 2019) but none concentrated in *P. marginatus*. We carried out the experiments to clone, identified the *FAR* genes from *P. marginatus*, and then found a set of suitable reference genes across all developmental stages. The candidate reference genes were derived from *P. marginatus* transcriptome data (X. Liang and Q. Chen, unpublished data) and include ribosomal protein L40 (*RPL40*), 18S ribosomal RNA (*18SrRNA*), beta actin (β*-actin*), beta tubulin (β*-TUB*), ADP-ribosylation factor 1 (*ARF1*), elongation factor 1 beta (*EF1-*β), and glyceraldehyde-3-phosphate dehydrogenase (*GAPDH*). The objective of this work was to select suitable reference genes to normalize the transcription of wax biosynthesis-related genes in different developmental stages of *P. marginatus*, while fed on resistant and susceptible cassava cultivars.

## Materials and Methods

### Mealybug

**Fig. 1.**
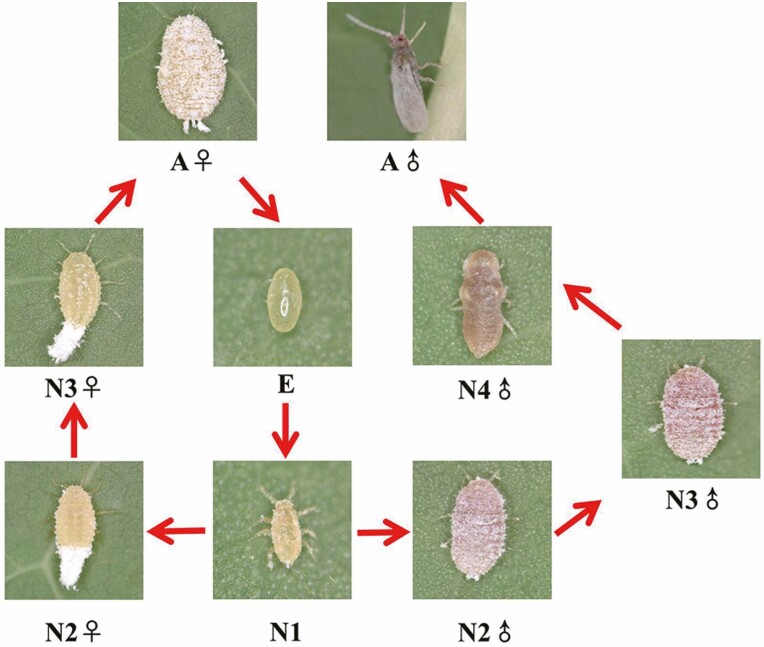
Life cycle of *Paracoccus marginatus*. E, egg; N1, sex-undifferentiated first-instar nymphs; N2, second-instar nymphs; N3, third-instar nymphs; N4, fourth-instar nymphs; A, adults.

*Paracoccus marginatus* were provided by the Environment and Plant Research Institution, Chinese Academy of Tropical Agriculture Sciences. Mealybugs were maintained on potato spouts (*Potato tuberosum* L.) supported with soil in plastic containers as a growing media (Nagrare et al. 2019). The insects were kept at 25 ± 1°C and 60–70% relative humidity (RH) under a 14 L: 10 D photoperiod in artificial climate chambers. The biological cycle of *P. marginatus* consists of four instars in the females and five in the males, which is N1 (sex-undifferentiated nymphs), male and female second-instar nymphs (N2♂ and N2♀, respectively), male and female third-instar nymphs (N3♂ and N3♀, respectively), male fourth-instar nymphs (N4♂), and male and female adults (A♂ and A♀), respectively (Fig. 1). Three biological replicates were collected for each active stage of *P. marginatus*, and the insect numbers for each replicate were listed as: 100 of N1, 30 of N2 (♂ or ♀), 30 of N3 (♂ or ♀), 30 of N4 (♂), and 30 of adults (♂ or ♀); in addition, male and female insects were separated. All samples were ground in liquid nitrogen and stored at –80°C until RNA extraction.

### Cultivation of Cassava Cultivars

Mealybug-susceptible cassava cultivars SC205 and mealybug-resistant cassava cultivars C1115 (Wang et al. 2018) were supplied by National Cassava Germplasm Nursery of China, CATAS. The stem segments with at least three eyes were vertically planted in pots (33 cm of diameter, 25 cm of height) containing 5 kg of well-mixed soil (soil: peat: perlite = 1:1:1) and grown in the greenhouse (28 ± 2°C and 14 L: 10 D photoperiod).

### Candidate Reference Genes and Target Genes

Seven candidate genes, *RPL40*, *18SrRNA*, β*-actin*, β*-TUB*, *ARF1*, *EF1-*β, and *GAPDH*, and two target genes, *PmFAR1* and *PmFAR2*, were used for performing RT-qPCR. Sequences of reference genes were also acquired by using homologous gene sequences from other mealybug species as query in blast searches against the *P. marginatus* transcriptome database (data not shown). The primers were designed by Oligo 6 and Primer Premier 5, and the detailed information is listed in Table 1.

**Table 1.** Primer information and PCR efficiency of the reference and target genes

Gene name	Primer sequence (5′–3′)	Product size (bp)	Tm (°C)	Efficiency (%)	*R* ^2^
CDS cloning					
* PmFAR1*	AAGGAGACGATGGAGGTAAT	1,638	56.8	Not tested	Not tested
	GCTATGGTTCCAATAGGGTA		57.2		
* PmFAR2*	TTTGTCAACCACCGAGATCA	1,640	56.7	Not tested	Not tested
	AAGCATCAGTTTTGAAAATACCTA		55.8		
Candidate reference gene					
* RPL40*	F: TGGACAAGGCACCAAGCG	226	57.6	97.65	0.9905
	R: ATGAAGGGTAGATTCTTTCTGGATA		58.3		
* 18SrRNA*	F: TCAAGACATGGTCGGAAGA	242	57.9	103.54	0.9871
	R: GGGAGGCATTGCTGGTA		57.2		
β*-actin*	F: CATCCTGCGTTTGGATTTAG	144	59.5	98.71	0.9926
	R: TCCAAAGCAACATAGCACAAT		57.7		
β*-TUB*	F: GTGGCGTTGTATGGTTCG	123	56.8	99.34	0.9784
	R: CGGTATGGGTACTTTACTTATCTC		57.6		
* ARF1*	F: AAGGAGGAGCCGCATCA	203	60.3	102.18	0.9796
	R: GCATCCAAACCCACCATTA		60.1		
* EF1-*β	F: CCTGAGCCTATCGTTTGC	240	58.8	97.56	0.9788
	R: CGCTTTCATGTCGGTTTC		59.5		
* GAPDH*	F: TCAAAACATCATCCCCGCAGCC	129	60.3	100.24	0.9882
	R: CGTCAAGTCGACGACCGAGACGT		59.2		
Target genes for validation					
* PmFAR1*	F: TATTCACGCAAATGGCAACG	109	58.9	99.37	0.9849
	R: AGGCTTCTTCAGTAAATCACAGC		58.5		
* PmFAR2*	F: TCATATTTCACGTCGCCGCTAG	212	61.2	97.82	0.9943
	R: GGCTTCCCTCCAGTTCATCG		60.7		

### RNA Extraction and cDNA Synthesis

Total RNA was extracted using Trizol reagent (Sangon, Shanghai, China) according to the manufacturer’s instructions. The quality and quantity of the extracted RNA were measured with a Thermo Scientific NanoDrop 2000 Spectrophotometer (Thermo Fisher Scientific Inc., Waltham, MA). The 260/280 nm absorption ratio for all samples was between 1.80 and 2.10, which indicates that RNA quality and purity were acceptable. After that, RNA was first treated with gDNA Eraser (TaKaRa, Shiga, Japan) to remove genomic DNA contamination. Then, 1.0 μ g of total RNA was used for first-strand cDNA synthesis by PrimeScript RT Reagent Kit (TaKaRa) according to the manufacturer’s recommendations. Finally, the cDNAs were stored at –80°C. Three biological replicates (three RNA and cDNA samples) are needed in each life stage of *P. marginatus* for evaluating the stability of candidate reference genes.

### RT-qPCR

RT-qPCR reactions were performed on a Roche LightCycler96 Real-Time PCR System (Roche Diagnostics Ltd, Rotkreuz, Switzerland). PCR reactions consisted of 7 µl of ddH_2_O, 10 μl of 2× SYBR Premix Ex TaqTM (TaKaRa), 0.5 μl of each specific forward and reverse primers (10 μmol/L), and 2 μl of first-strand cDNA (fivefold diluted cDNA) in a final volume of 20 μl. The RT-qPCR conditions include the following: an initial denaturation for 2 min at 95°C followed by 40 cycles of denaturation at 95°C for 5 s and annealing at 60°C for 30 s, and a final elongation step of 72°C for 60 s. For the melting curve analysis, a dissociation step cycle (65°C for 5 s, and then an increase of 0.5°C every 10 s up to 95°C) was used. The reactions were set up in 96-well PCR plates in triplicate (technical replicates) for each biological sample. A threefold serial dilution of cDNA (1/3, 1/9, 1/27, 1/81, 1/243, 1/729, 1/2,187, and 1/6,561) was used to construct the standard curves for each gene, and PCR grade water replacing the same volume of cDNA template was used as negative control (no template control, also three replicates). The RT-qPCR efficiency (*E*) was calculated according to the following equation: *E* = 10^(–1/slope)–1)^ × 100. To avoid between-run variations, three independent technical replicates were performed for each sample for all experiments. Furthermore, the between-run correction factor, which is derived from the target quantities which are calculated from the quantification threshold, PCR efficiency, and observed Cq value, is used for removal of between-run variation in a multiplate qPCR experiment according to Ruijter et al. (2015).

### Data Analysis for Transcription Stability of Candidate Reference Genes

The raw data of quantification cycle (Cq) were obtained from each experiment. The stability of each gene was analyzed by softwares like geNorm, NormFinder, BestKeeper, and the ΔCq method. The comparative ΔCq method is used to identify useful reference genes by comparing the relative transcription of gene pairs within each sample. The most stable reference genes are associated with the lowest SDs of ΔCq when these genes are compared with the other reference genes (Morales et al. 2016). The geNorm algorithm calculates an expression stability value (*M*) for each gene based on geometric mean (GM). Firstly, the sample with minimum Cq was used as the calibrator with a set value of 1.0, and that of the other samples is 2^−ΔCq^, where ΔCq = Cq value of the gene in the selected sample − minimum Cq value of the corresponding gene in the experiment. After these data were imported into the geNorm program, and the *M* values were calculated, usually the smaller the *M* value is, the more stable the reference gene would be; in addition, *M* = 1.5 is the upper limit, and only genes with *M* value less than 1.5 are considered to be relatively stable (Vandesompele et al. 2002, Niu et al. 2012). Additionally, the geNorm program can calculate the optimal number of reference genes for valid normalization of RT-qPCR, and the method is as follows: first select the two reference genes with the best stability to calculate the normalization factor (NF), and then add the next gene one by one for calculation. If NF value is less than 0.15, it corresponds to the number of used reference genes, and if more than one combination has NF values lower than 0.15, then the minimum number of reference genes are usually chosen (Zheng et al. 2019), as an additional reference gene will not significantly improve the normalization (Yang et al. 2014). To input the transcription data for NormFinder analysis, the Cq values were also transformed to quantities using the formula 2^ΔCq^. NormFinder can directly generate the stability value (SV) via variance analysis of each reference gene, and then rank them according to their SV values to determine the most stable gene (Morales et al. 2016). The original Cq data of each gene can be directly input to the Bestkeeper program. BestKeeper can calculate transcription stability of each reference gene and inter-reference gene relations through numerous pairwise correlation analyses. The most stable reference genes are considered to be those with the highest Pearson correlation coefficients (*r*) or lowest SDs (Niu et al. 2012). Finally, we comprehensively compared and ranked the candidate genes using a web-based analysis tool RefFinder (http://www.leonxie.com/referencegene.php), which can integrate ΔCq, Bestkeeper, Normfinder, and geNorm algorithms (Pan et al. 2015), to obtain the optimal reference gene among the candidates.

### Gene Cloning and Sequence Analysis

By using FAR sequences from other mealybug species as query in blast searches against the transcriptomic database (data not shown) of *P. marginatus*, two fragments coding *PmFAR1* and *PmFAR2* were found. The open reading frames (ORFs) of *PmFAR1* and *PmFAR2* were predicted using ORF Finder (https://www.ncbi.nlm.nih.gov/orffinder/), and then primers were designed to clone the CDS of *PmFAR1* and *PmFAR2* (Table 1), assuming that the ORF and CDS fragments have the identical sequences. After the PCR products were sequenced, and the CDS of *PmFAR1* and *PmFAR2* were translated into amino acid using Protparam (http://web.expasy.org/protparam/). Signal peptide cleavage sites were predicted using SignalP. Known *FAR* gene sequences from other scale insects were subjected to multiple sequence alignment using the ClustalW mode of BioEdit (version 7.2.6), and the Conserved Domain Database (https://www.ncbi.nlm.nih.gov/Structure/cdd) was used to predict the presence of conserved motifs on the FAR gene sequences. Using these sequence analysis procedures, we verified the identity of the CDS of *PmFAR1* and *PmFAR2*. PCR reactions were performed at 95°C for 3 min, followed by 40 cycles of 15 s at 95°C and 30 s at 58°C. The PCR products were purified with a gel extraction kit (TAKARA, Dalian, China) and linked with plasmid pMD19-T vector (TAKARA) to form a recombinant plasmid, and then transferred into competent cell *DH5a* (TAKARA) for sequencing.

### Validation of Reference Gene Selection in Different Life Stages of *P. marginatus*

To examine the reliability of the selected reference genes, the transcript levels of *PmFAR1* and *PmFAR2* in different life stages of *P. marginatus* (from first nymph to adult, male and female insects were separated; the insect numbers for each RNA sample were listed as follows: 100 of N1, 30 of N2 [♂ or ♀], 30 of N3 [♂ or ♀], 30 of N4 [♂], and 30 of adults [♂ or ♀]) were analyzed by using the most stable or unstable reference genes that determined above. The relative transcription of the *PmFAR1* and *PmFAR2* gene was calculated by the 2^–ΔΔCq^ method (Pfaffl 2001). The relative transcription of *FAR* gene in each developmental stage was calculated relative to the transcription of *PmFAR1* at first-instar nymph (N1)

### Analysis the Transcription of *FAR* Gene When *P. marginatus* Fed on Host Plant

The same amount of mealybugs (100 of N1, 30 of N2 [male and female mixed], 30 of N3 [male and female mixed], 30 of N4 [male], and 30 of female adults) were starved for 12 h and then inoculated on fresh leaflets of SC205 (susceptible cassava cultivars) and C1115 (resistant cassava cultivars), after fed for 48 h under reared condition until the insects were sampled. Each treatment was replicated three times. RNA extraction, RT-qPCR (used the optimal reference gene combination), and data analysis were performed as described above. While *P. marginatus* fed on the susceptible cassava cultivar SC205, the transcription of PmFAR1 and PmFAR2 was defined as 1.0, while the transcription of the rest of the samples was presented as the change fold relative to it.

## Results

### Expression Profiling of Selected Reference Genes in *P. marginatus*

**Fig. 2.**
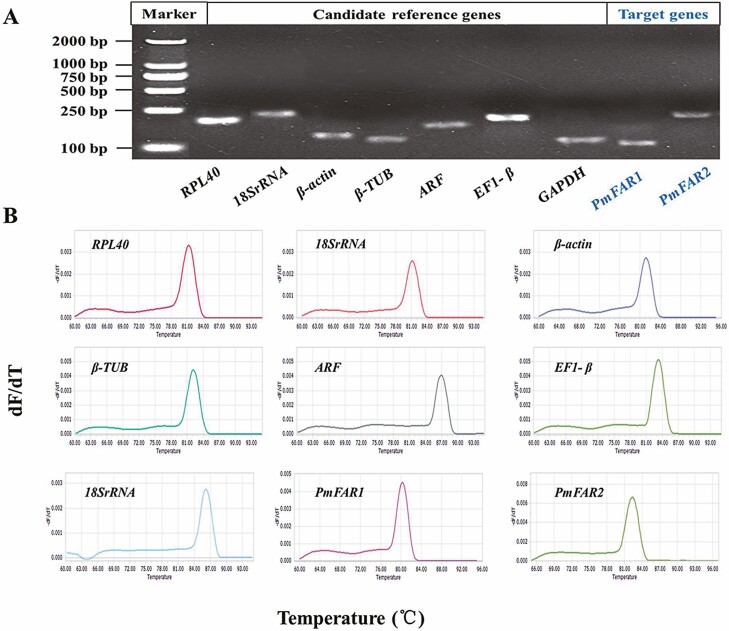
Specificity of the seven selected candidate reference genes and two target FAR genes. (A) The PCR products of the nine genes were resolved on a 2% agarose gel. (B) Specificity of RT-qPCR of these nine genes. Dissociation curves with single peaks were generated from all amplicons.

The PCR products of the seven candidate reference genes together with the two target genes (*PmFAR1* and *PmFAR2*) showed single bands on 2% agarose gel electrophoresis (Fig. 2A), and the nucleic acid sequences were all identical to those derived from the transcriptome database (Supp Table S1 [online only]). In addition, only one gene-specific peak and absence of primer dimer peaks in the RT-qPCR melting curves suggest that each of the primer pairs amplified a unique product (Fig. 2B). These results showed that the amplified sequences of these nine genes were accurate and the primer specificity was eligible for further analysis. In addition, the PCR efficiency ranged from 97.56 to 103.54% and the correlation coefficient (*R*^2^) of all genes was higher than 0.9784 (Table 1).

**Fig. 3.**
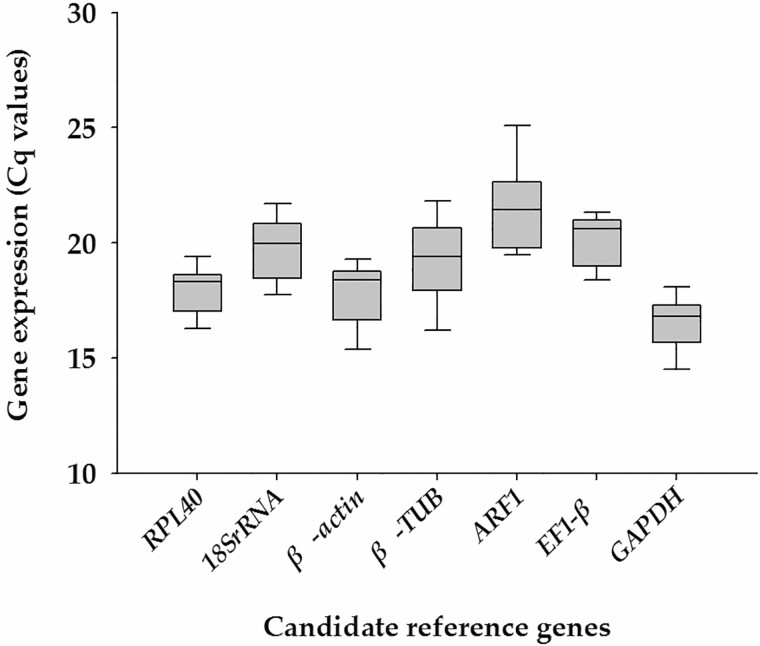
Expression profiles of the seven reference genes of *P. marginatus*. The expression of candidate reference genes was documented in Cq value. The median is represented by the line in the box. The interquartile rangy is bordered by the upper and lower edges, which indicate the 75th and 25th percentiles, respectively.

Gene transcription analyses of the seven candidate reference genes exhibited a narrow Cq range across all samples of *P. marginatus* from different developmental stages, with the median Cq values varying from 16.39 (*GAPDH*) to 21.45 (*ARF1*). Among these genes, *GAPDH* (median Cq = 16.39) was the most abundantly expressed gene, followed by *β-actin* (median Cq = 17.48). The least expressed gene was *ARF1* (median Cq = 21.45) (Fig. 3; Supp Table S2 [online only]). β-Actin had the least variable transcription reflected by the lowest SD value (0.96), while β-TUB had the most variable transcription (SD = 1.31) (Supp Table S2 [online only]).

### Stability of Candidate Reference Genes in Different Developmental Stages

#### ΔCq Analysis

The stability rankings of the candidate genes, from the most stable (the lowest SD) to the least stable (the highest SD) gene, was as follows: β*-actin* > *RPL40* > *GAPDH* > *ARF1* > *18SrRNA* > *EF1-*β > β*-TUB* (Table 2; Supp Tables S2 and S3 [online only]).

**Table 2.** Stability ranking of the seven reference genes using four different algorithms

ΔCq		geNorm		NormFinder		BestKeeper		RefFinder	
Gene	SD	Gene	*M*	Gene	SV	Gene	r	Gene	GM
β*-actin*	0.95	β*-actin*	0.29	*GAPDH*	0.31	*GAPDH*	0.972	β*-actin*	3.15
*RPL40*	1.02	*RPL40*	0.31	β*-actin*	0.33	β*-actin*	0.961	*GAPDH*	3.46
*GAPDH*	1.03	*GAPDH*	0.32	*RPL40*	0.45	*EF1-*β	0.953	*RPL40*	3.59
*ARF1*	1.13	*18SrRNA*	0.39	*18SrRNA*	0.49	*RPL40*	0.942	β*-TUB*	4.03
*18SrRNA*	1.15	β*-TUB*	0.48	*EF1-*β	0.56	β*-TUB*	0.932	*EF1-*β	5.12
*EF1-*β	1.23	*EF1-*β	0.55	β*-TUB*	0.79	*ARF1*	0.904	*18SrRNA*	5.69
β*-TUB*	1.31	*ARF1*	0.69	*ARF1*	0.94	*18SrRNA*	0.877	*ARF1*	6.37

GM, geometric mean; M, average of expression stability values; *r*, Pearson correlation coefficient; SV, stability value.

#### GeNorm Analysis

Data from Table 2 show that all the *M* values of the seven candidate reference genes were below the threshold limit of *M* value = 1.5, indicating that all candidate genes had stable transcription. The stability rankings of the candidate genes, from the most stable (the lowest *M* value) to the least stable gene (the highest *M* value) were listed as follows: β*-actin* > *RPL40* > *GAPDH* > *18SrRNA* > β*-TUB* > *EF1-*β > *ARF1*.

#### NormFinder Analysis

The stability rankings of the candidate genes, from the most stable (the lowest SV) to the least stable (the highest SV) gene, were as follows: *GAPDH* > β*-actin* > *RPL40* > *18SrRNA* > *EF1-*β > β*-TUB* > *ARF1* (Table 2).

#### BestKeeper Analysis

The BestKeeper analyzes each gene’s transcription variability by calculating the Cq set SD and Pearson correlation coefficients (*r* values). The most stable reference genes are considered to be those with the highest *r* values or lowest SD. The stability rankings of candidate genes, from the most stable (the highest *r*) to the least stable (the lowest *r*) gene were as follows: *GAPDH* > β*-actin* > *EF1-*β > *RPL40* > β*-TUB* > *ARF1* > *18SrRNA* (Table 2; Supp Table S4 [online only]).

#### Comprehensive Analysis of RefFinder

According to RefFinder, the stability ranking, from the most (lowest GM value) to the least stable (highest GM value) across the developmental stages, was as follows: β*-actin* > *GAPDH* > *RPL40* > β*-TUB* > *EF1-*β > *18SrRNA* > *ARF1*. The most stable genes was β*-actin* (GM = 3.15), which was consistent with the rankings from ΔCq and NormFinder analysis, besides, the least stable gene was *ARF1* (GM = 6.37) (Table 2).

#### Optimal Number of Reference Genes for RT-qPCR Normalization

**Fig. 4.**
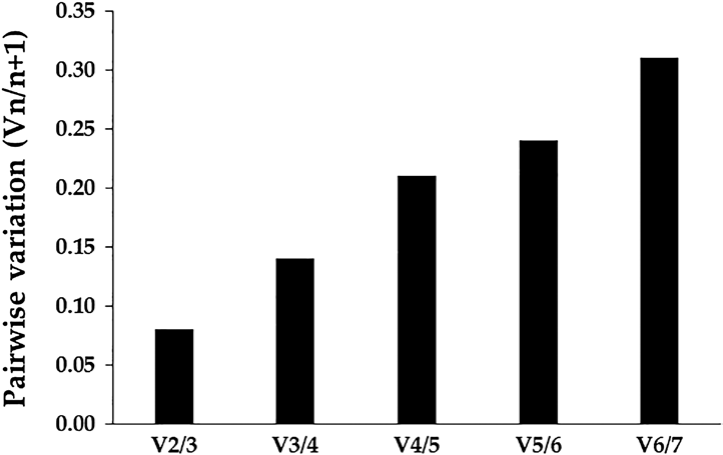
Optimal number of reference genes for normalization in RT-qPCR analysis of *P. marginatus* genes.

The geNorm program can provide the optimal number of reference genes under specific experimental condition through calculating the pairwise variation (*Vn*/*n* + 1) values. As shown in Fig. 4, *V*_2/3_ = 0.08 that was <0.15, therefore β*-actin* and *GAPDH* were considered to be the two most suitable reference genes for *P. marginatus* at different developmental stages.

### Sequence Analysis of *PmFAR1* and *PmFAR2*

**Fig. 5.**
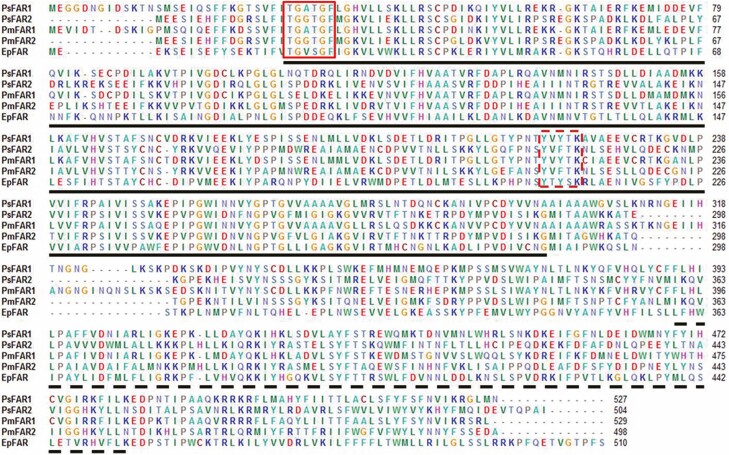
Multi sequencing alignment from FARs of *P. marginatus* and other scale insects, including *Ph. solenopsis* (*PsFAR1*, GenBank accession number: ANN23959.1; *PsFAR2*, GenBank accession number: ANN23960.1) and *E. pela* (*EpFAR*, GenBank accession number: AGK27745.1). Rossmann folding and FAR_C region were indicated in black lines and dashed lines, respectively. Two motifs TGXXGF and YXXXK were indicated in box and dotted box.

The CDS of *PmFAR1* and *PmFAR2* were 1,590 and 1,497 bp in length, encoding 529 and 498 amino acids, respectively (Supp Fig. S1 [online only]). In addition, both PmFAR1 and PmFAR2 were nonsecretory proteins since no signal peptide was found by SignalP 4.1. The result of conserved domain analysis showed that both PmFAR1 and PmFAR2 have a Rossmann folding and a FAR_C region (Fig. 5), which belonged to the FAR_C superfamily (Supp Fig. S2 [online only]). The multisequencing alignment of FAR amino acid sequences of *Ph. solenopsis* and *E. pela* showed that two highly conserved motifs were discovered as shown in Fig. 5, including a NADPH combining motif, TGXXGF, and an active site motif, YXXXK.

### Validation of Reference Gene Selection in Different Life Stages of *P. marginatus*

**Fig. 6.**
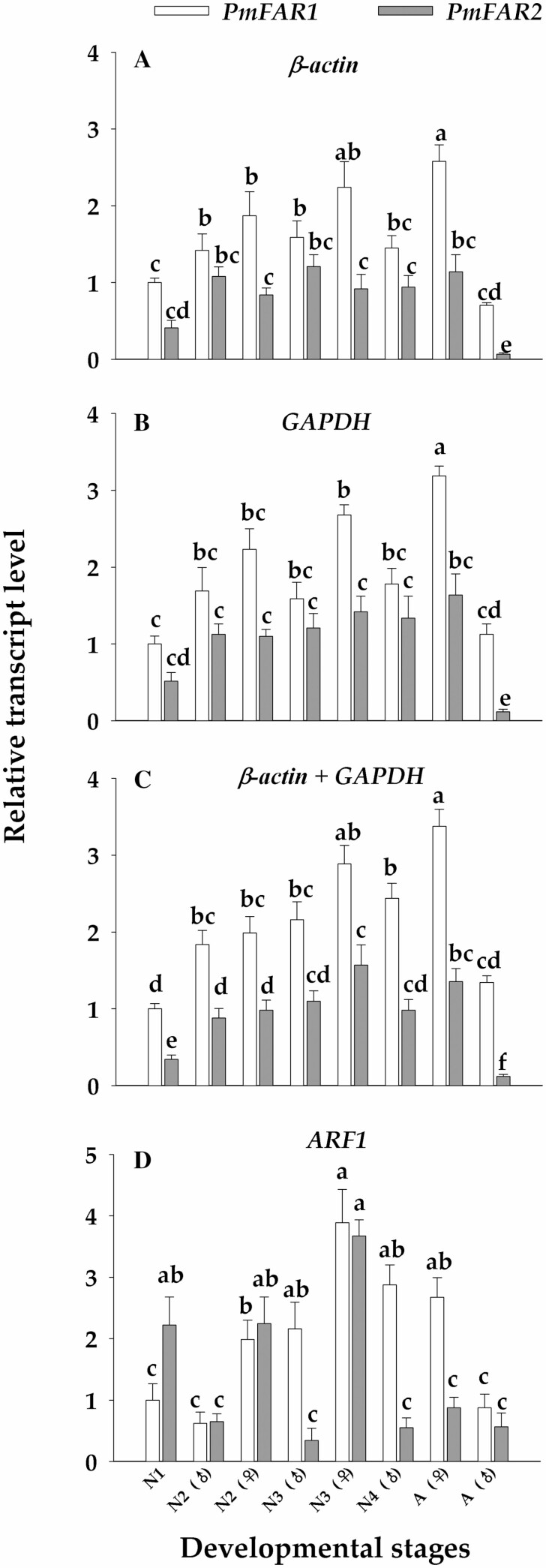
Relative transcript levels of *PmFAR1* and *PmFAR2* in different developmental stages of *P. marginatus*. The relative transcription of each gene in each developmental stage was calculated relative to the transcript level of *PmFAR1* at first-instar nymph (N1). Different letters above the SE bars indicate significant differences of transcript level based on the one-way analysis of variance followed by Duncan’s multiple range tests (*P* < 0.05). β*-Actin* (A), *GAPDH* (B), β*-actin* + *GAPDH* (C), and *ARF1* (D) were used as reference genes for evaluating the transcript level of *PmFAR1* and *PmFAR2.*

When the two best reference genes (β*-actin* and *GAPDH*) and their combination were used for normalization, similar transcription profiles were obtained for *PmFAR1* and *PmFAR1* in the different developmental stages of males and females. The transcript levels of *PmFAR1* and *PmFAR2* basically showed a distinct pattern—that is, the transcription increased from first nymph (N1) to third nymph (N3), and then decreased in the fourth nymph and adult (Fig. 6). In addition, the transcription of PmFAR1 was always higher than PmFAR2 in different life stages, however, significant difference could be observed while used the reference gene combination for normalization, while using single reference (β-actin or GAPDH) was not necessarily so (P < 0.05) (Fig. 6A and 6B). In addition, when the most unstable reference gene *ARF1* was used, some contrary results appeared, i.e., the transcriptions of *PmFAR2* in N1 and N2 female were higher than *PmFAR1*, besides, higher deviation was also observed (Fig. 6D).

### Transcription Analysis of *FAR* Gene When *P. marginatus* Fed on Host Plant

**Fig. 7.**
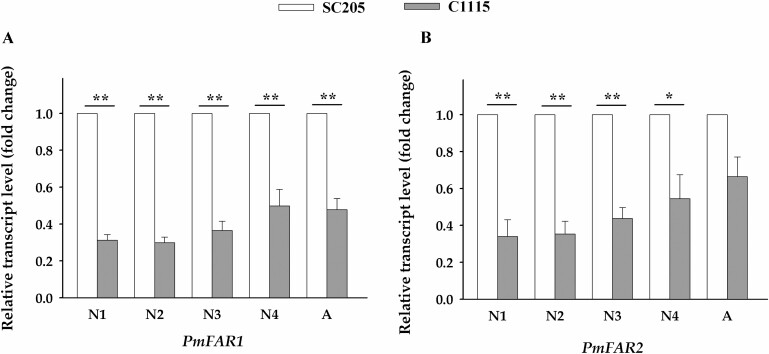
Reference gene combination β*-actin* + *GAPDH* was used to analyze the expression of *PmFAR1* and *PmFAR2* of *P. marginatus* in different developmental stages. While *P. marginatus* fed on the susceptible cassava cultivar SC205, the transcription of *PmFAR1* and *PmFAR2* was defined as 1.0, while the transcription of the rest of the samples was presented as the change fold relative to it. Independent-samples *T*-test (SPSS, version 19.0) was used for statistical analysis; asterisk indicate significant different: *P < 0.05, **P < 0.01.

At different developmental stages of *P. marginatus* fed on resistant cassava cultivar C1115 and susceptible cassava cultivars SC205, the changes in the transcription of *PmFAR1* and *PmFAR2* were analyzed by the reference gene combination (β*-actin* + *GAPDH*). The results showed that the transcription of these two genes in any life stages (except *PmFAR2* in adults) of *P. marginatus* fed on C1115 was significantly lower than those fed on SC205 (Fig. 7).

## Discussion

Selection and validation of reference gene are quite important for reliable RT-qPCR normalization. Use of a single reference gene without validation of its stability can lead to considerable error in calculating gene transcription (Yang et al. 2015). According to Morales et al. (2016), there are over 40 arthropod species that have been investigated for reference gene identification and selection under diverse experimental conditions. To the best of our knowledge, literatures about reference gene selections in mealybug species were all related to *Ph. solenopsis* (Arya et al. 2017, Singh et al. 2018, Zheng et al. 2019) (Table 3); however, there are no molecular-based studies conducted yet in *P. marginatus*. This study, for the first time, evaluated and identified the most stable reference genes for the validation of gene transcriptions in *P. marginatus*. Among the seven selected candidate reference genes, we found β*-actin* and *GAPDH* were the most stable genes.

**Table 3.** Comparison of reference genes for *Paracoccus marginatus* and *Phenacoccus solenopsis* under different experimental conditions

Cases	Scientific name^a^	Candidate gene	Experimental condition	Best reference genes	Reference
1	*Phenacoccus solenopsis* (Hemiptera: Pseudococcidae)	*α-Tubulin*, β*-Tubulin*, *Actin*, *Rpl32*, *GAPDH*, *SDHA*	Developmental stages Field distribution Host plant-feeding assays Temperature treatments	β*-Tubulin* β*-Tubulin* β*-Tubulin*, *α-Tubulin* *α-Tubulin*, *RPL32*	Arya et al. (2017)
2	*Phenacoccus solenopsis* (Hemiptera: Pseudococcidae)	*EF1-*β, *EF1-δ*, *RPL32*, *RPL40*, *RP18S*, *RP28S*, *STX16*, *TUB-α*, *TBP and UB-E2*	Developmental stages Sex-dimorphic development	*EF1-*β, *RPL32*	Zheng et al. (2019)
3	*Phenacoccus solenopsis* (Hemiptera: Pseudococcidae)	*Actin*, *28srRNA*, *EF*, *VATPase*, *TUB*, *UBIQE3*, *MYOSIN*, *TATA*, *TFIID*, *SOD*, *GST*, *RPS39*, *ANNEXIN*, *SDHA*	Developmental stages Starvation stress RNA interference	*GST*, *Actin*, *TFIID*, *SDHA*, *28srRNA*	Singh et al. (2019)
4	*Phenacoccus solenopsis* (Hemiptera: Pseudococcidae)	Not mentioned	Spirotetramat treatment	β*-Tubulin*	Li et al. (2016)
5	*Paracoccus marginatus* (Hemiptera: Pseudococcidae)	*RPL40*, *18SrRNA*, β*-actin*, β*-TUB*, *ARF1*, *EF1-*β, *GAPDH*	Developmental stages Fed on susceptible and resistant cassava cultivars	β*-actin*, *GAPDH*	This study

Ranking of stable reference genes largely depended on the different software. Among the seven candidate genes, β*-actin* was selected as the most stable reference genes by both the ΔCq method and geNorm, while the NormFinder and BestKeeper analysis results showed *GAPDH* was the most stable gene. These differences may result from the differences in the statistical algorithms among the different analysis tools. For examples, BestKeeper analyzes the stability among candidate reference genes. NormFinder and the ΔCq method focus on pairwise variation between two reference genes, and then determine the stability of one of them (Zheng et al. 2019). According to RefFinder, an analysis tool which can integrate the results of these statistical algorithms and produce a comprehensive ranking of all candidate reference genes (Yang et al. 2015), β*-actin* and *GAPDH* were considered to be the most suitable reference genes. These two genes were stable in the host plant-feeding assays, and these results were not identical to formal studies about another mealybug species *Ph. solenopsis*, in which Tubulin or ribosomal protein genes were usually supposed to be the best candidates (Table 3) (Li et al. 2016, Arya et al. 2017). Also, this study supports the point that there is no single ‘universal’ reference gene even in closely related species, as suitable reference gene depends on specific experimental condition.

In present study, the CDS of two FAR genes *PmFAR1* and *PmFAR2* in *P. marginatus* were successfully cloned and characterized. Like genes from other scale insect species (i.e., *Ph. solenopsis* and *E. pela*), both *PmFAR1* and *PmFAR2* had conservative motifs belonging to FAR_C superfamily, indicating the potential function in wax biosynthesis (Chacón et al. 2013). With suitable reference gene, the transcription and function investigations of FAR *gene* could be appropriately conducted. In addition, while using stable reference gene (no matter single one or combination) to normalize transcription of *PmFAR1* and *PmFAR2* in different developmental stages of males and females insects, similar transcription profiles were obtained. However, the significant differences of transcription between *PmFAR1* and *PmFAR2* in certain life stage were not fully revealed by single reference gene (β*-actin* or *GAPDH*), while these subtle differences could be observed by using two stable reference genes (β*-actin* + *GAPDH*). In fact, for most of the cases, two or even more validated stable reference genes are often recommended for accurate RT-qPCR normalization (Thellin et al. 1999, Bustin et al. 2005, Morales et al. 2016). Altogether, the transcription of *PmFAR1* gradually increases from nymph to female adult, while the transcription of *PmFAR2* shows the same pattern before the third nymph stage and then significantly decreases in the fourth nymph and male adults. This phenomenon can make sense because *P. marginatus* are typical sex-dimorphic insects, and the male adult produces few waxes (Miller and Miller 2002). By contrast, the transcription of the two *FAR* genes in *Ph. solenopsis* was significantly higher in the first-instar nymphs than in other developmental stages (Li et al. 2016), and very low transcription was detected in *FAR2* gene of these two mealybug male adults, which indicates that the transcription pattern of *FAR* gene shows a species-dependent manner.

Here, we found that at different developmental stages of *P. marginatus* fed on resistant cassava cultivars, the transcriptions of both *PmFAR1* and *PmFAR2* were significantly reduced with the exception of *PmFAR2* in female adult. This phenomenon indicated that the suppression of *PmFAR1* and *PmFAR2* by resistant cassava plant would probably account for the decrease in the survival rate or fecundity of *P. marginatus* that we found in formal studies (Chen et al. 2020). We assumed that the inhibition of *FAR* genes may be a promising way in controlling *P. marginatus*, and, to a certain extent, these results may explain the resistant mechanism of cassava to mealybug; but this needs further investigation.

In conclusion, we selected the suitable reference genes for analyzing the transcriptions of *PmFAR1* and *PmFAR2* in *P. marginatus* under condition of host plant-feeding assay, and found the reduction of transcriptions of both PmFAR1 and PmFAR2 while mealybug fed on resistant cassava cultivars. This study might probably help in better understanding the molecular mechanism of cassava resistance to mealybug. However, there is a lack of clear evidence that *FAR* gene is directly related to mealybug wax accumulation on the epidermis. Future studies could manipulate transcription of the *FAR* gene (i.e., RNA interference or gene editing) and detect whether the wax contents and components are changed accordingly; those definitive gene-to-phenotype relationships will specify the function of *FAR* gene in mealybug species.

## Supplementary Material

ieab072_suppl_Supplementary_Materials
