# Supplementary material for: Reference Gene Selection for Analyzing the Transcription Patterns of Two Fatty Acyl-CoA Reductase Genes From Paracoccus marginatus (Hemiptera: Pseudococcidae)
Source: J Insect Sci. 2021 Oct 4;21(5):11. doi: 10.1093/jisesa/ieab072 (PMC8489056; doi:10.1093/jisesa/ieab072)
Supplement: ieab072_suppl_Supplementary_Materials [file ieab072_suppl_supplementary_materials.docx]

**Table S1** PCR product sequencing result showing the amplified sequences of the given primers.

| **Gene name** | | **Primer sequence（5’-3’）** | | **Product size (bp)** | **Sequences of the PCR products （5’-3’）^1^** |
| --- | --- | --- | --- | --- | --- |
| **7 Candidate reference gene** | | | |  |  |
| *RPL40* | F: TGGACAAGGCACCAAGCG | | | 226 | >*RPL40*  TGGACAAGGCACCAAGCGAAAATGCAGATTTTCGTGAAAACCCTCACGGGTAAGACCATCACCCTCGAGGTCGAGGCCTCCGATACCATCGAAAATGTGAAGGCCAAGATCCAGGACAAAGAGGGTATCCCTCCGGACCAGCAGAGGTTGATCTTCGCTGGCAAGCAGCTCGAGGATGGTCGCACTTTGTCCGACTACAATATCCAGAAAGAATCTACCCTTCATC |
|  | R: ATGAAGGGTAGATTCTTTCTGGATA | | |  |  |
| *18SrRNA* | F: TCAAGACATGGTCGGAAGA | | | 242 | >*18SrRNA*  TCAAGACATGGTCGGAAGAATTTATACTAATAGCTGAATCAATTATAACAGATAATGAAGGATATACTGAAGATAGGTCGTTGTAAAGTTTCTCCTCTACTGAAAATTCGACTAGCATTTTAGTTAATGGTGTTACGCTGGTGACAGTTTTTATTAAAGGCGATGCAGATACTAAAATTATCGATGCACCTGGATCAAAAATGGAGATATGTTCGATACACGACATACCAGCAATGCCTCCC |
|  | R: GGGAGGCATTGCTGGTA | | |  |  |
| *β-actin* | F: CATCCTGCGTTTGGATTTAG | | | 144 | >*β-actin*  CATCCTGCGTTTGGATTTAGCTGGTCGTGATTTAACCGACTATTTAATGAAAATCCTCACTGAAAGAGGTTATAGCTTTACCACAACTGCCGAAAGAGAAATCGTACGTGATATTAAAGAAAAATTGTGCTATGTTGCTTTGGA |
|  | R: TCCAAAGCAACATAGCACAAT | | |  |  |
| *β-TUB* | F: GTGGCGTTGTATGGTTCG | | | 123 | >*β-TUB*  GTGGCGTTGTATGGTTCGACTACGGTATCCGATACTTTAGGTGATGGTACGACTGAGTAAGTATTCATTATTCTATCGGGATATTCTTCGCGGATTTTTGAGATAAGTAAAGTACCCATACCG |
|  | R: CGGTATGGGTACTTTACTTATCTC | | |  |  |
| *ARF1* | F: AAGGAGGAGCCGCATCA | | | 203 | >*ARF*  AAGGAGGAGCCGCATCAGTCGTGTCTCGTGTCCAGCCGAACGCGGAGGGTCGACGCCTCGCCGCTGGATTACTTGCTAACCACGTCAACGCATATCATCCGAAGTTGAAGAAATAATACCACAATGGGTCTGACGATCAGTACCATGTTGACTCGCCTTTTTGGCAAAAAACAAGTACGTATTCTAATGGTGGGTTTGGATGC |
|  | R: GCATCCAAACCCACCATTA | | |  |  |
| *EF1-β* | F: CCTGAGCCTATCGTTTGC | | | 240 | >*β-*EF1  CCTGAGCCTATCGTTTGCAAGAAACCACAAGTTGCCAAACCACCAGCAAAGAAAGAAGACGATGACGACGTTGATCTTTTCGGTTCTGAATCTGAGGAGGAAGACGAAGAAGCTAAACGAATCAAAGAAGAACGAGTAGCTGCTTATGCTGCTAAAAAATCCAAAAAACCAGTATTAATCGCAAAATCAAACATAATATTGGATGTTAAACCTTGGGACGATGAAACCGACATGAAAGCG |
|  | R: CGCTTTCATGTCGGTTTC | | |  |  |
| *GAPDH* | F: TCAAAACATCATCCCCGCAGCC | | | 129 | > *GAPDH*  TCAAAACATCATCCCCGCAGCCACTGGCGCCGCCAAGGCCGTCGGCAAGGTCATCCCATCTCTGAACGGCAAACTCACCGGTATGGCTTTCCGTGTCCGGTCGCCAACGTCTCGGTCGTCGACTTGACG |
|  | R: CGTCAAGTCGACGACCGAGACGT | | |  |  |
| **2 target genes for validation** | | | |  |  |
| *PmFAR1* | | | F: TATTCACGCAAATGGCAACG | 109 | > *PmFAR1*  TATTCACGCAAATGGCAACGGAATTAACCAAAATTCTCTCAAATCAAAATCAGAAGACTCGAAAAATATCACCGTCTATAACTACAGCTGTGATTTACTGAAGAAGCCT |
|  |  |  | R: AGGCTTCTTCAGTAAATCACAGC |  |  |
| *PmFAR2* | | | F: TCATATTTCACGTCGCCGCTAG | 212 | > *PmFAR2*  TCATATTTCACGTCGCCGCTAGTGTCAGATTCGACGATCCGATTCACGAAGCGATCATCATCAATACCAGAAGTACCAGAGAGGTTGTAACTTTGGCGAAGGAAATCAAGAATATTGCTGTTTTAGTCCACGTATCCACAACGTACTGTAATTCGTACCGTAAAGTAGTCGAAGAGAAGATATACCCAGCTCCGATGAACTGGAGGGAAGCC |
|  |  |  | R: GGCTTCCCTCCAGTTCATCG |  |  |

^1^ The corresponding forward and reverse primers located in the sequences were marker with green and yellow shadows, respectively.

**Table S2** Analysis of Cq values of seven candidate housekeeping genes in all samples.

| **Gene** | **N^1^** | **Median** | **Lowest** | **Lower**  **quartile** | **Higher**  **quartile** | **Highest** | **Mean** | **SD^2^** |
| --- | --- | --- | --- | --- | --- | --- | --- | --- |
| *RPL40* | 48 | 18.05 | 16.27 | 16.68 | 18.34 | 18.83 | 18.42 | 1.02 |
| *18SrRNA* | 48 | 19.96 | 17.73 | 18.12 | 20.56 | 21.08 | 20.13 | 1.15 |
| *β-actin* | 48 | 17.48 | 15.38 | 15.81 | 17.92 | 18.48 | 17.56 | 0.95 |
| *β-TUB* | 48 | 19.34 | 17.65 | 18.23 | 19.85 | 20.52 | 19.37 | 1.31 |
| *ARF1* | 48 | 21.45 | 19.47 | 19.86 | 21.86 | 22.11 | 21.38 | 1.13 |
| *EF1-β* | 48 | 20.16 | 18.38 | 18.74 | 20.91 | 21.33 | 20.26 | 1.23 |
| *GAPDH* | 48 | 16.39 | 14.52 | 15.08 | 17.15 | 17.42 | 16.41 | 1.03 |

^1^ N: number of Cqs

^2^ SD: standard deviation.

| Gene | *RPL40* | | *18SrRNA* | | *β-actin* | | *β-TUB* | | *ARF1* | | *EF1-β* | | *GAPDH* | |
| --- | --- | --- | --- | --- | --- | --- | --- | --- | --- | --- | --- | --- | --- | --- |
|  | **Mean**  **ΔCq** | **SD** | **Mean**  **ΔCq** | **SD** | **Mean**  **ΔCq** | **SD** | **Mean**  **ΔCq** | **SD** | **Mean**  **ΔCq** | **SD** | **Mean**  **ΔCq** | **SD** | **Mean**  **ΔCq** | **SD** |
| *RPL40* | *0.00* | *0.00* | -1.78 | *1.41* | 0.71 | 0.78 | -1.35 | *1.91* | -3.21 | *1.21* | -2.15 | *1.87* | 1.65 | *0.78* |
| *18SrRNA* | 1.78 | *1.31* | 0.00 | *0.00* | 2.49 | *1.02* | 0.44 | *1.46* | -1.43 | *1.34* | -0.37 | *1.64* | 3.44 | *1.37* |
| *β-actin* | -0.71 | *1.08* | -2.49 | *1.51* | 0.00 | *0.00* | -2.06 | *1.58* | -3.92 | *1.12* | -2.86 | 1.26 | 0.94 | *1.54* |
| *β-TUB* | 1.35 | *1.12* | -0.44 | *1.33* | 2.06 | *1.33* | 0.00 | *0.00* | -1.86 | *1.09* | -0.80 | *1.05* | 3.00 | *0.76* |
| *ARF1* | 3.21 | *1.34* | 1.43 | *1.15* | 3.92 | *0.98* | 1.86 | *1.26* | 0.00 | *0.00* | 1.06 | *0.97* | 4.86 | *1.63* |
| *EF1-β* | 2.15 | *1.05* | 0.37 | *1.34* | 2.86 | *1.31* | 0.80 | *1.43* | -1.06 | *1.51* | 0.00 | *0.00* | 3.80 | *1.13* |
| *GAPDH* | -1.65 | *1.22* | -3.44 | *1.28* | -0.94 | *1.24* | -3.00 | *1.56* | -4.86 | *1.64* | -3.80 | *1.84* | 0.00 | *0.00* |
| Mean SD |  | 1.02 |  | 1.15 |  | 0.95 |  | 1.31 |  | 1.13 |  | 1.23 |  | 1.03 |

**Table S3** Pairwise comparison of candidate housekeeping genes based on the analysis by the comparative ΔCq method.

**Table S4** Statistical analyses of nine selected candidate reference genes based on the BestKeeper algorithm.

|  | ***RPL40*** | ***18SrRNA*** | ***β-actin*** | ***β-TUB*** | ***ARF1*** | ***EF1-β*** | ***GAPDH*** |
| --- | --- | --- | --- | --- | --- | --- | --- |
| N | 48 | 48 | 48 | 48 | 48 | 48 | 48 |
| GM [Cq] | 18.46 | 20.08 | 17.57 | 19.52 | 21.71 | 20.02 | 16.65 |
| AM [Cq] | 18.42 | 20.13 | 17.56 | 19.37 | 21.38 | 20.26 | 16.41 |
| Min [Cq] | 16.27 | 17.73 | 15.38 | 17.65 | 19.47 | 18.38 | 14.52 |
| Max [Cq] | 18.83 | 21.08 | 18.48 | 20.52 | 22.11 | 21.33 | 17.42 |
| SD [± Cq] | 1.02 | 1.15 | 0.95 | 1.31 | 1.13 | 1.23 | 1.03 |
| CV [% Cq] | 4.53 | 2.98 | 3.76  76 | 5.91 | 5.06 | 3.94 | 4.13 |
| [r] | 0.942 | 0.877 | 0.961 | 0.932 | 0.904 | 0.953 | 0.972 |
| *P*-value | 0.001 | 0.001 | 0.001 | 0.001 | 0.001 | 0.001 | 0.001 |

N: number of Cqs; GM [Cq]: geometric means of the quantification cycle (Cq); AM [Cq]: the arithmetic mean of Cq; Min [Cq] and Max [Cq]: the extreme values of Cq; SD [± Cq]: the standard deviation of the Cq; CV [% Cq]: the coefficient of variance expressed as a percentage at the Cq level; [r]: Pearson correlation coefficient.


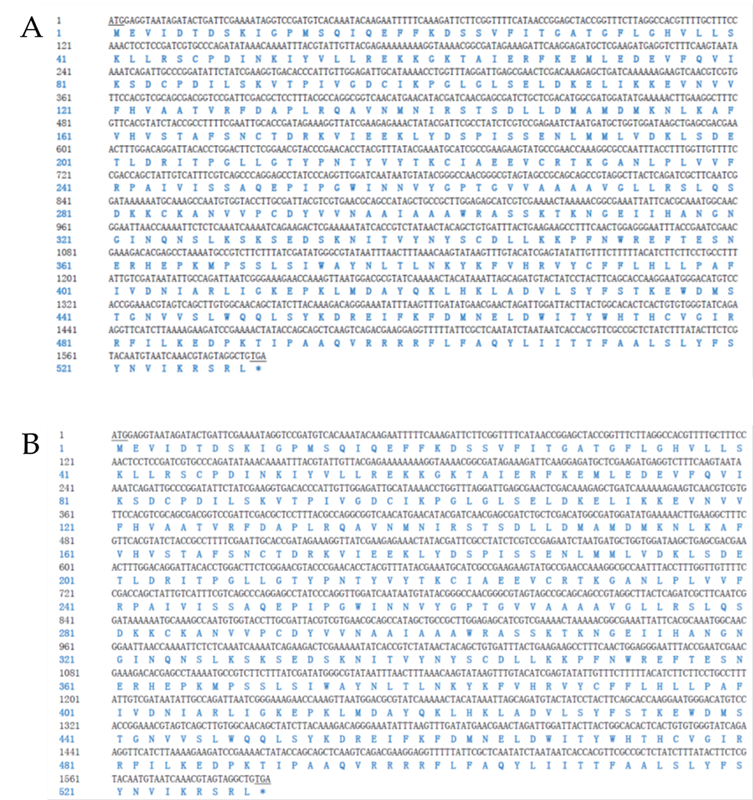


**Figure S1** Coding sequence and deduced amino acid sequence of *PmFAR1* (**A**) and *PmFAR2* (**B**). The underline denotes the initiation and the translation stop codon


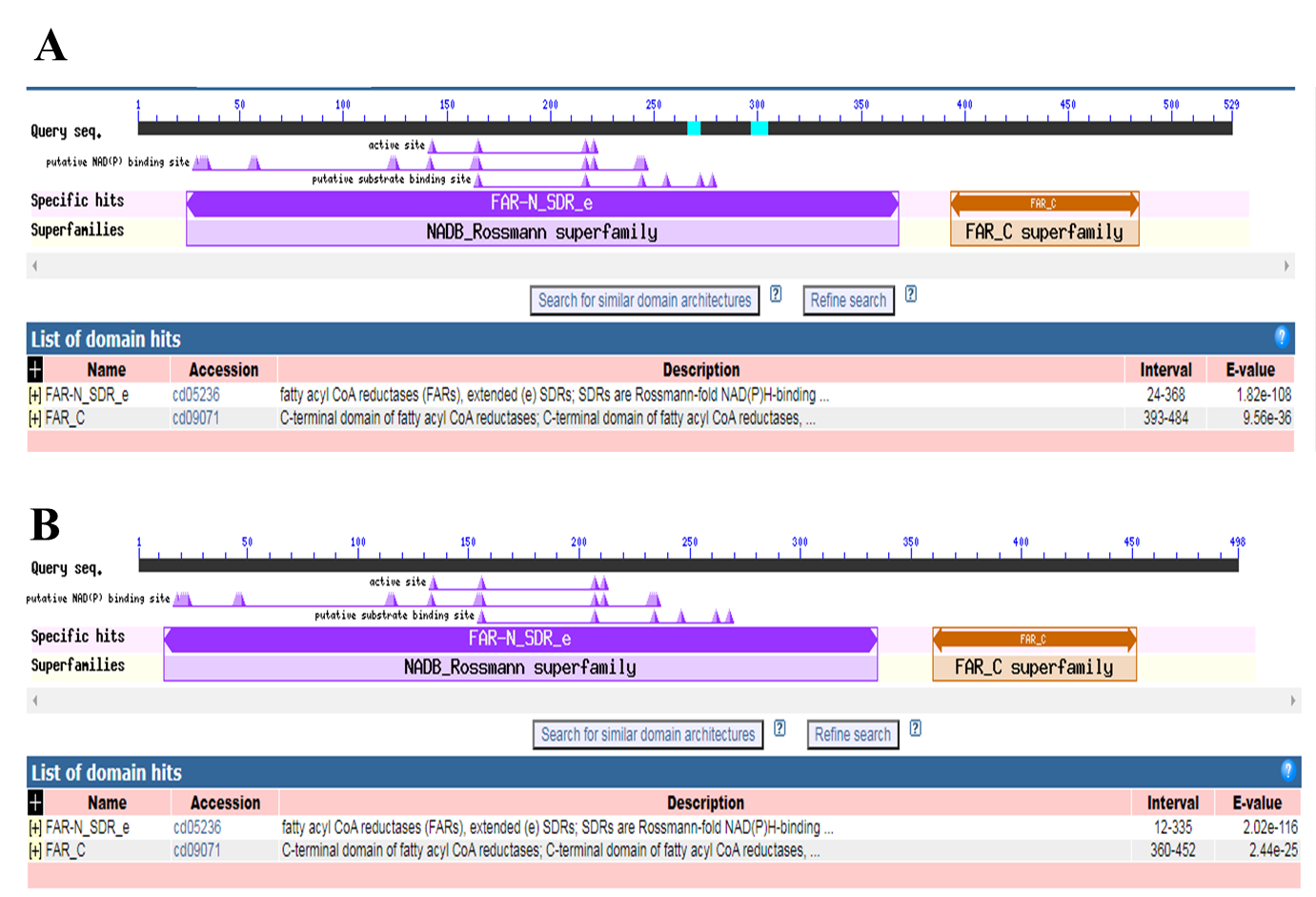


**Figure S2** Prediction of conserved amino acid domain for *PmFAR1* (**A**) and *PmFAR2* (**B**). The prediction results were derived from the Conserved domain database (https://www.ncbi.nlm.nih.gov/Structure/cdd)
